# Immune Checkpoint Inhibitors in Gastrointestinal Cancers: Current Evidence and Future Directions

**DOI:** 10.32604/or.2025.065818

**Published:** 2025-10-22

**Authors:** Takeshi Toyozumi, Hideaki Shimada, Hisahiro Matsubara

**Affiliations:** 1 Department of Frontier Surgery, Graduate School of Medicine, Chiba University, Chiba, 260-0856, Japan; 2 Department of Gastroenterological Surgery and Clinical Oncology, Toho University Graduate School of Medicine, Tokyo, 143-8541, Japan

**Keywords:** Immune checkpoint inhibitors, gastrointestinal tumor, conversion surgery

## Abstract

Cancer immunotherapy has long been established as an important treatment option for cancers. In particular, Immune Checkpoint Inhibitor (ICI) has been reported to be effective against various gastrointestinal cancers (esophageal cancer, gastric cancer, colorectal cancer); however, the treatment phase in which ICI should be used and how it should be incorporated into the treatment strategy vary depending on the cancer type being treated. Multiple clinical trials and basic research on ICIs are currently underway, and new insights from these results will continue to change the clinical treatment strategy of gastrointestinal cancers. While it is desirable to have an increasing number of treatment strategy options for gastrointestinal cancers, it is necessary to organize increasingly complex treatments and select more appropriate ICI-based treatments. In addition, as gastrointestinal cancers are being controlled through multidisciplinary treatment using ICI-based treatment, local control by conversion surgery is becoming an important treatment option. We may soon see an era in which gastrointestinal cancers can be systematically controlled with ICI-based treatment, while difficult-to-control lesions can be removed by conversion surgery. In this review, we summarize the evidence of ICI-based treatment for gastrointestinal cancers and provide an overview of the treatment strategies currently underway.

## 1 Introduction

Cancer immunotherapy has gained attention in recent years as a new paradigm in tumor treatment, and Immune Checkpoint Inhibitor (ICI) has brought about groundbreaking progress [1,2]. Immune checkpoints are naturally occurring control mechanisms that prevent excessive activation of T cells, but it has been revealed that tumor cells exploit this mechanism to evade immune surveillance [3,4].

In particular, “Programmed Death-1 (PD-1)” is an important molecule that suppresses the immune response of T cells, and its function was elucidated by Dr. Tasuku Honjo and others, leading to the development of immunotherapy targeting the PD-1/Programmed Death Ligand-1 (PD-L1) pathway [5,6]. In addition, “Cytotoxic T-Lymphocyte–Associated Protein 4 (CTLA-4)” is another critical immune checkpoint molecule that regulates early T cell activation, and its inhibitory role was first elucidated by Dr. James P. Allison and others [7], ultimately contributing to the development of immune checkpoint inhibitors targeting the CTLA-4 pathway in cancer immunotherapy. Compared with conventional chemotherapy and molecular targeted therapy, this ICI has shown promising therapeutic effects, especially against recurrent and refractory solid tumors, and clinical applications have been established in non-small cell lung cancer [8], malignant melanoma [9], renal cell cancer [10], and other cancers ahead of other fields [11,12].

Meanwhile, ICI treatment for gastrointestinal cancers (esophageal cancer (EC), gastric cancer (GC), and colorectal cancer (CC)) has also progressed, with many clinical trials being conducted. Gastrointestinal cancers are a global public health issue due to their high incidence and mortality rates, and the development of new treatments is urgently needed due to the limitations of standard treatments. It is speculated that the expression level of PD-1 and PD-L1 in tumor tissue may be related to the prognosis of patients and the therapeutic effect of ICI, and various studies have been conducted. Many studies have also focused on the concentration of PD-L1 in serum, but no consensus has yet been reached [13]. There have been many reports of the search for biomarkers to predict the therapeutic effect of ICI in the field of gastrointestinal cancer [14–16]. However, although there are several biomarker candidates, there is still no established biomarker based on consensus.

Although immune checkpoint inhibitors have shown sustained therapeutic effects in some patients, predictors of efficacy and resistance mechanisms have not been fully elucidated [17]. In addition to monotherapy, combination therapies with chemotherapy, radiation therapy, molecular targeted therapy, and gut microbiota adjustment are being explored, and their efficacy and safety are being evaluated [18,19]. Various genes and proteins have been reported as potentially controlling the tumor immune environment, but it is difficult to say that conclusive evidence has been established [20,21].

While highly effective in cancer treatment, ICI can cause autoimmune reactions called immune-related Adverse Events (irAE). According to a systematic review in 2025, approximately 40% of patients treated with ICI experienced some kind of irAE, of which approximately 20% experienced severe adverse events of grade 3 or higher [22]. In particular, the incidence of irAE was as high as 45.7% in combination therapy including CTLA-4 inhibitors, and the risk of severe irAE tends to increase. In addition, a large-scale international survey in 2024 reported that irAE, such as myocarditis, severe skin reactions (SCARs), myositis, and pneumonia, showed high mortality rates, with irAE-related mortality rates of 19.2% for myocarditis and 12.3% for SCARs [23]. These findings indicate the importance of early detection and appropriate management of irAE in ICI treatment.

This paper focuses on recent trends in ICI treatment for gastrointestinal cancers and aims to contribute to the development of future treatments by outlining the latest findings on clinical trial results and treatment strategies.

## 2 Esophageal Cancer

### 2.1 Established Clinical Treatment Based on ICI for Esophageal Cancer

**Figure 1:**
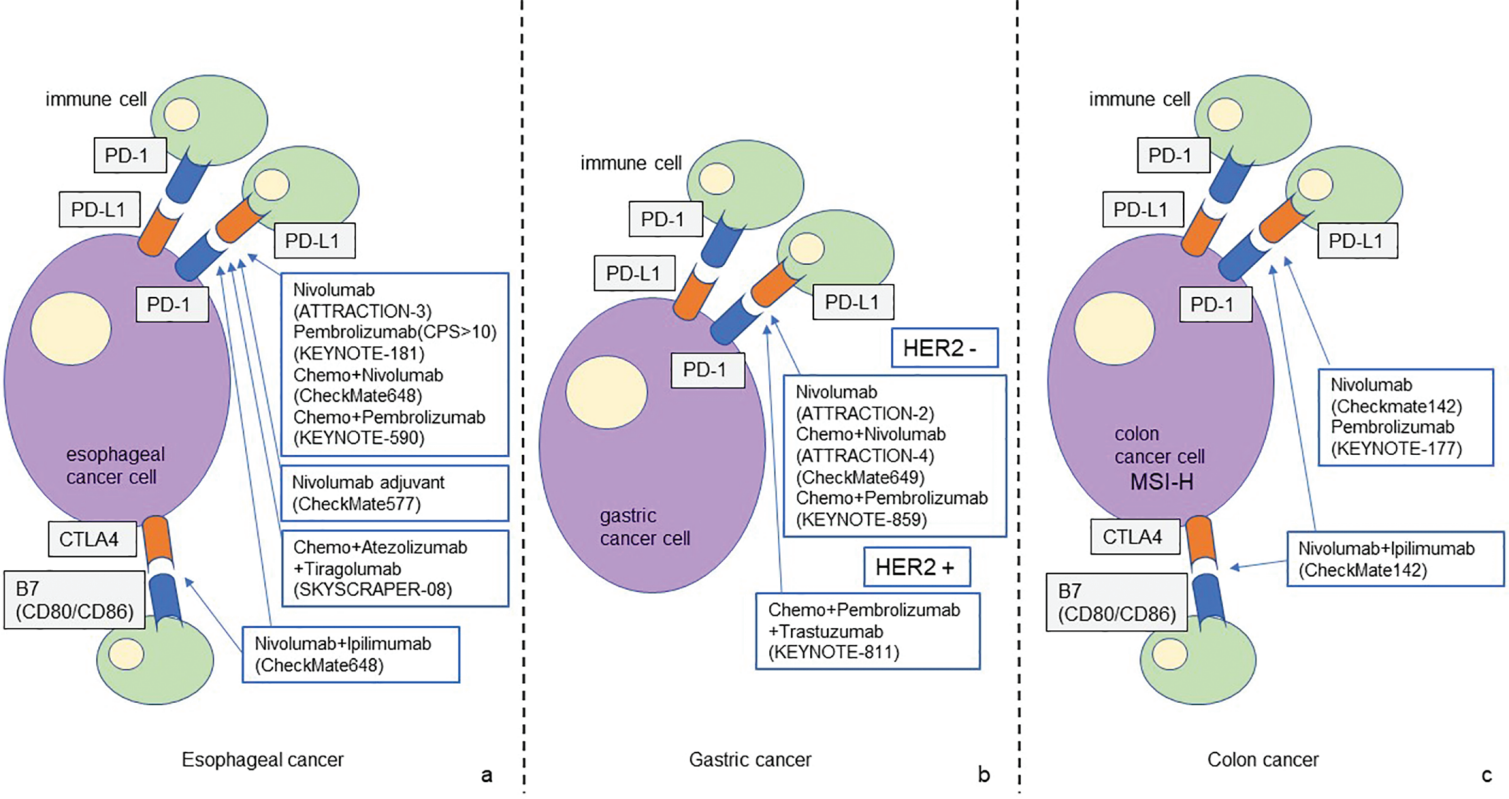
The schematic figure of ICI-based treatment strategy for gastrointestinal cancer

Esophageal cancer is often difficult to treat curatively, and cancer immunotherapy is being actively introduced as new evidence emerges. The main autoimmune antibody drugs are “anti-PD-1 antibodies” and in recent years, “anti-Cytotoxic T-Lymphocyte Antigen-4 (CTLA-4) antibodies” have also been introduced. This chapter details the results of important clinical trials that show the forefront of cancer immunotherapy for esophageal cancer from an academic perspective. The recent clinical trials in ICI treatment for esophageal cancer are detailed below and summarized in Table 1 and Fig. 1a.

**Table 1:** The recent clinical trials in ICI treatment for esophageal cancer

Trial	Phase	Treatment target	Intervention	Control	Endpoint	Result
ATTRACTION-3	III	Progressive/ recurrent	Nivolumab monotherapy	Chemo	OS	Positive
KEYNOTE-181	III	Progressive/ recurrent	Pembrolizumab monotherapy	Chemo	OS (CPS > 10)	Positive
KEYNOTE-590	III	Progressive/ recurrent	Pembrolizumab + chemo	Chemo	OS, PFS	Positive
CheckMate648	III	Progressive/ recurrent	Nivolumab + chemo	Chemo	OS, PFS	Positive
		Progressive/ recurrent	Nivolumab + Ipilimumab	Chemo	OS, PFS	Positive
CheckMate-577	III	Adjuvant	Nivolumab monotherapy	Placebo	DFS	Positive
JCOG2206	III	Adjuvant	Nivolumab monotherapy	No treatment	RFS	In progress
			TS-1 monotherapy			
Keystone-001	II	Neo adjuvant	Pembrolizumab + chemo	–	MPR rate	–
Keystone-002	III	Neo adjuvant	Pembrolizumab + chemo	Chemo	EFS	In progress
HCHTOG1909	III	Neo adjuvant	Toripalimab + chemo	Chemo	EFS	In progress
JCOG1804E	I	Neo adjuvant	Nivolumab + chemo	–	Tolerability	–
LEAP-014	III	Progressive/ recurrent	Lenvatinib + Pembrolizumab + chemo	Pembrolizumab + chemo	OS	In progress
SKYSCRAPER-08	III	Progressive/ recurrent	Tiragolumab + Atezolizumab + chemo	Chemo	OS, PFS	positive
JCOG2311	II	Progressive/ recurrent	Radiation + Nivolumab + Ipilimumab	–	PFS	In progress

Note: TS-1, Tegafur Gimeracil Oteracil Potassium; OS, Overall Survival; CPS, Combined Positive Score; PFS, Progression Free Survival; DFS, Disease-Free Survival; RFS, Recurrence-Free Survival; MPR, Major Pathological Response; EFS, Event-Free Survival.

The figure shows the ICI-based treatment strategies for gastrointestinal cancer that have been established to date. PD-1/PD-L1 and CTLA4 are the main treatment targets for esophageal cancer (a). PD-1/PD/L1 is the main treatment target for gastric cancer, and new treatment strategies for HER2-positive gastric cancer are currently being developed (b). The efficacy of ICI-based treatment for MSS colon cancer has not been elucidated. Treatment targeting PD-1/PD-L1 and CTLA4 has been established for MSI-H colon cancer (c).

In the treatment of esophageal cancer, the effectiveness of ICI for unresectable advanced recurrent cancer has been widely proven. Based on the results of CheckMate-577, Nivolumab monotherapy is recommended as adjuvant chemotherapy after radical resection, followed by neoadjuvant chemoradiotherapy [24], but adjuvant chemotherapy after neoadjuvant chemotherapy is currently being verified. New developments include exploring the effectiveness of ICI as neoadjuvant chemotherapy, and combinations of ICI with new drugs and with radiation therapy.

ATTRACTION-3 study is a phase III randomized clinical trial comparing nivolumab (anti-PD-1 antibody) monotherapy with standard chemotherapy (paclitaxel or docetaxel) in patients with recurrent or progressive esophageal squamous cell carcinoma. In this study, patients who progressed after first-line treatment were included, and Overall Survival (OS) was set as the primary endpoint. As a result, the median OS in the nivolumab group was 11.1 months, which was significantly longer than the 8.5 months in the chemotherapy group (Hazard Ratio (HR) = 0.77, 95% confidence interval (CI): 0.62–0.96, *p* = 0.019). The 1-year survival rate was 43% in the nivolumab group and 30% in the chemotherapy group (statistical significance not stated), demonstrating the superiority of immunotherapy. In addition, in terms of safety, Treatment-Related Adverse Events (TRAEs) were reduced in the nivolumab group (63% vs. 18%), and patients’ quality of life (QOL) was also maintained [25]. Based on the results of ATTRACTION-3, nivolumab monotherapy can be used after second-line chemotherapy for esophageal cancer treatment. The latest European Society for Medical Oncology (ESMO) clinical practice guidelines recommend nivolumab monotherapy in the second-line treatment of patients with esophageal squamous cell carcinoma after platinum-based and fluoropyrimidine-based chemotherapy (I, A; MCBS 3) [26]. Furthermore, according to the 2022 clinical guidelines of the Japan Esophageal Society, nivolumab is strongly recommended as a second-line treatment for advanced esophageal squamous cell carcinoma [27].

KEYNOTE-181 is a phase III clinical study comparing pembrolizumab (anti-PD-1 antibody) monotherapy with standard chemotherapy (paclitaxel, docetaxel, or irinotecan) in patients with recurrent or metastatic esophageal cancer. In this study, patients with PD-L1 expression level (Combined Positive Score (CPS) ≥ 10) were the primary evaluation subjects, and OS was the primary endpoint. PD-L1 expression was assessed at baseline tumor samples (biopsies or resections from primary or metastatic lesions) before the start of treatment. CPS was defined as (PD-L1-positive tumor cells tumor-infiltrating immune cells)/number of viable tumor cells × 100. The results showed that in patients with PD-L1 expression and CPS ≥ 10, the median OS in the pembrolizumab group was 9.3 months, significantly longer than that in the chemotherapy group (6.7 months) (HR = 0.69, 95% CI: 0.52–0.93, *p* = 0.0074). Grade 3–5 TRAEs occurred in 18.2% of patients in the pembrolizumab group and 40.9% of patients in the chemotherapy group. The therapeutic effect was particularly notable in patients with esophageal squamous cell carcinoma, suggesting that immunotherapy may be a new treatment option [28]. Based on the results of the KEYNOTE-181 study, nivolumab monotherapy can be used after second-line chemotherapy for the treatment of esophageal squamous cell carcinoma with high CPS expression (CPS ≥ 10).

KEYNOTE-590 study is a phase III clinical study evaluating the combination of pembrolizumab and chemotherapy (cisplatin + 5-FU) in patients with advanced or recurrent esophageal cancer. OS and Progression Free Survival (PFS) were set as primary endpoints. As a result, the median OS in the pembrolizumab combination group was 12.4 months, which was significantly longer than the chemotherapy alone group at 9.4 months (HR = 0.62, 95% CI: 0.49–0.78, *p* < 0.0001). PFS was also significantly improved, with the median PFS in the pembrolizumab combination group being 6.3 months, which was significantly longer than the chemotherapy alone group at 5.8 months (HR = 0.65, 95% CI: 0.55–0.76, *p* < 0.0001). The effect was particularly notable in patients with high PD-L1 expression (CPS ≥ 10) (OS, HR = 0.57, 95% CI: 0.43–0.75, *p* < 0.0001, PFS, HR = 0.51, 95% CI: 0.41–0.65, *p* < 0.0001). Efficacy was confirmed in both esophageal squamous cell carcinoma and adenocarcinoma, suggesting that combination therapy with chemotherapy may become a new standard of care [29]. Grade ≥3 TRAEs occurred in 72% of patients in the pembrolizumab plus chemotherapy group and 68% in the placebo plus chemotherapy group.　Based on the results of the KEYNOTE-590 study, combination therapy of pembrolizumab and chemotherapy (cisplatin + 5-FU) is recommended as first-line chemotherapy for esophageal cancer treatment.

CheckMate648 is a Phase III clinical trial evaluating the combination therapy of nivolumab + chemotherapy and nivolumab + ipilimumab (anti-CTLA-4 antibody) in patients with previously untreated advanced esophageal squamous cell carcinoma. The primary endpoints were OS and PFS in the PD-L1 high expression group (Tumor Proportion Score (TPS) ≥ 1%). As a result, the median OS in the nivolumab + chemotherapy group was 13.2 months and in the nivolumab + ipilimumab group was 12.7 months, both of which were significantly longer than the chemotherapy alone group (10.7 months) (nivolumab + chemotherapy, HR = 0.74, 99.1% CI: 0.58–0.96, *p* = 0.002, nivolumab + ipilimumab, HR = 0.78, 98.2% CI: 0.62–0.98, *p* = 0.01). The median PFS of the nivolumab + chemotherapy group was 6.9 months, significantly longer than that of the chemotherapy alone group (4.4 months) (HR = 0.65, 98.5% CI: 0.46–0.92, *p* = 0.002), but the PFS of the nivolumab + ipilimumab group was 4.0 months, and no additional benefit was observed over the chemotherapy alone group (HR = 1.02, 98.5% CI: 0.73–1.43, *p* = 0.90). The incidence of grade 3 or 4 TRAEs was 47% in patients receiving nivolumab + chemotherapy, 32% in patients receiving nivolumab + ipilimumab, and 36% in patients receiving chemotherapy alone. Interestingly, despite the OS benefit observed with nivolumab + ipilimumab, PFS was not improved compared to chemotherapy alone, suggesting a potential delayed treatment effect or pseudoprogression typical of ICI. The effect was particularly notable in patients with high PD-L1 expression, demonstrating the usefulness of combined immunotherapy in extending OS. In the safety evaluation, treatment-related adverse events, including immune-related adverse events, were within the predictable and manageable range, and tolerability was deemed acceptable [30]. Based on the results of the CheckMate648 trial, nivolumab and chemotherapy (cisplatin + 5-FU) or nivolumab + ipilimumab therapy is recommended as first-line chemotherapy for esophageal cancer, but the decision should be made taking into account a comprehensive range of factors, including the patient’s condition [26].

However, there is no conclusion as to whether chemotherapy + PD-1 antibody or nivolumab + ipilimumab should be selected for first-line chemotherapy of esophageal squamous cell carcinoma, and there is no evidence to serve as a basis for selection. Similarly, there is no specific consensus on whether the PD-1 antibody to be used in combination with chemotherapy should be nivolumab or pembrolizumab, and it is entirely up to the clinician’s judgment. In addition to collecting real-world data, we hope to generate new evidence.

Additionally, the CheckMate577 study is a phase III clinical trial evaluating the efficacy of nivolumab as adjuvant therapy in patients with esophageal or esophagogastric junction cancer who underwent curative surgery after preoperative chemoradiotherapy. Both adenocarcinoma and squamous cell carcinoma were recruited for this study. The primary endpoint was Disease-Free Survival (DFS). As a result, the median DFS in the nivolumab group was 22.4 months, which was significantly longer than that in the placebo group (11.0 months) (HR = 0.69, 96.4% CI: 0.56–0.86, *p* < 0.001). Grade 3 or 4 TRAEs occurred in 13% of patients in the nivolumab group and 6% in the placebo group. TRAEs led to discontinuation of the study regimen in 9% of patients in the nivolumab group and 3% of patients in the placebo group. The effect of reducing the risk of postoperative recurrence was demonstrated, confirming the potential of immunotherapy as an adjuvant therapy [24]. Adjuvant chemotherapy using nivolumab is applicable to non-pathological Complete Response (pCR) esophageal cancer and esophagogastric junction cancer that have undergone radical resection after preoperative chemoradiotherapy, but there is no evidence of adjuvant chemotherapy for esophageal cancer that has undergone radical resection after preoperative chemotherapy. In Japan, a prospective multi-institutional randomized trial is underway in which patients with esophageal squamous cell carcinoma who have undergone preoperative adjuvant chemotherapy (FP (Fluorouracil+Cisplatin) or DCF (Fluorouracil+Cisplatin+Docetaxel)) are divided into three groups: a group that receives adjuvant nivolumab therapy, a group that receives adjuvant TS-1 (Tegafur Gimeracil Oteracil Potassium), and a group that has not undergone treatment (JCOG2206, SUNRISE trial) [31], and the results are expected to be promising. However, JCOG2206, the SUNRISE trial, examined patients who received neoadjuvant chemotherapy, not neoadjuvant chemoradiotherapy. Although the study subjects are in line with the results of the JCOG1109 trial [32], a previous study in Japan, the trial results should be interpreted with caution regarding this difference. Similarly, in some areas, postoperative adjuvant chemotherapy using ICI is being performed for patients with esophageal squamous cell carcinoma who have undergone radical resection after neoadjuvant chemotherapy. It should be recognized that there is no established evidence for this approach.

### 2.2 Future Perspectives of ICI for Esophageal Cancer

The future prospects for cancer immunotherapy using ICI in the field of esophageal cancer can be divided into the introduction of ICI in perioperative chemotherapy and the development of ICI combination therapy aimed at increasing the therapeutic effect for unresectable advanced cancer. This chapter provides an overview of representative clinical trial efforts in each area.

Examples of the introduction of ICI in perioperative chemotherapy include the Keystone-001 and Keystone-002 trials. The Keystone-001 trial is a phase II trial targeting cases of resectable esophageal squamous cell carcinoma who underwent robot-assisted esophagectomy after three cycles of pembrolizumab combination chemotherapy as neoadjuvant chemotherapy. The primary endpoints were safety and Major Pathological Response (MPR) rate. As a result, the MPR rate was 72%, the pCR rate was 41%, and the 2-year OS was 91%, and the 2-year DFS was 41%, confirming good treatment outcomes. In addition, there were no Grade 3 or higher adverse events or delays in the timing of surgery during neoadjuvant therapy [33]. These findings show preoperative pembrolizumab plus chemotherapy is a promising therapeutic strategy for resectable esophageal squamous cell carcinoma.

Based on these results, Keystone-002, a multicenter, prospective, randomized, phase III clinical trial, is underway. Patients with cT1N2M0 or cT2-3N0-2M0 esophageal squamous cell carcinoma are randomly assigned to either neoadjuvant chemotherapy with pembrolizumab plus chemotherapy or neoadjuvant chemoradiotherapy. The primary endpoint is Event Free Survival (EFS). The secondary endpoints are 1-, 3-, and 5-year OS and DFS, short-term outcomes, and QOL [34]. It is expected that the results of the Keystone-002 trial will make neoadjuvant chemotherapy with pembrolizumab plus chemotherapy a new treatment option for esophageal cancer.

Additionally, a phase III trial of neoadjuvant chemotherapy in combination with toripalimab, a new PD-1 antibody drug, is being conducted in China (HCHTOG1909). The primary endpoint is EFS. The secondary endpoints include pCR, DFS rate, OR rate, R0 resection rate, MPR, Adverse Events (AEs), complication rate and QOL [35]. Results of the HCHTOG1909 trial are also pending.

In Japan, a phase I trial was conducted to explore the tolerability and efficacy of nivolumab plus chemotherapy (FP, DCF, FLOT (Fluorouracil + Levofolinate + Oxaliplatin + Docetaxel)) as neoadjuvant chemotherapy (JCOG1804E, FRONTiER trial) [36]. As a result, the tolerability of neoadjuvant chemotherapy combined with nivolumab was confirmed, and new clinical trials are expected to be planned.

A retrospective study comparing preoperative adjuvant chemoradiotherapy and preoperative ICI-based treatment for locally advanced esophageal cancer has been conducted in China. According to this study, preoperative ICI-based treatment significantly extended 2-year DFS and OS after surgery compared to preoperative chemoradiotherapy [37]. This result suggests that preoperative ICI-based treatment may become a treatment option for locally advanced esophageal cancer.

As part of the development of ICI combination therapy aimed at increasing the therapeutic effect for unresectable advanced cancer, a randomized open-label phase III trial, LEAP-014, is currently underway, which combines the antiangiogenic agent lenvatinib with pembrolizumab and chemotherapy [38]. In addition, in the SKYSCRAPER-08 study, a phase III trial that investigated the efficacy of combination therapy with the anti-T cell Immunoreceptor with Ig and ITIM domains (TIGIT) antibody tiragolumab and the PD-L1 inhibitor atezolizumab plus chemotherapy, it has been reported at the conference presentation that combination therapy of tiragolumab, atezolizumab, and chemotherapy significantly extends PFS and OS.

In Japan, a phase 2 trial is currently underway to explore the PFS extension effect of combination therapy combining nivolumab + ipilimumab therapy with short-term radiation therapy (JCOG2311, ART NOUVEAU trial) [39]. The ICI and radiation therapy combination therapy is considered to be one of the main topics in future esophageal cancer treatment, and the results of the trial are attracting attention.

An interesting report from Japan has been published regarding the relationship between the timing of nivolumab infusions and therapeutic efficacy for esophageal squamous cell carcinoma. They divided esophageal cancer patients into two groups: the infusion of nivolumab before 13:00 was set as ‘early in the day’, and that after 13:00 was set as ‘late in the day’. As a result, OS, PFS, and objective response rate (ORR) of patients who received the first dose in the early group were significantly superior to those of patients in the late group. It may be that the activation of tumor immune cells also varies circadianly, and there may be an optimal time period for infusion of ICI [40].

## 3 Gastric Cancer

### 3.1 Established Clinical Treatment Based on ICI for Gastric Cancer

Cancer immunotherapy for gastric cancer has been highlighted as an important treatment option for unresectable advanced gastric cancer, recurrent gastric cancer, and esophagogastric junction adenocarcinoma. This chapter academically explains the contents and results of important clinical trials of cancer immunotherapy for gastric cancer and provides references. The recent clinical trials in ICI treatment for gastric cancer are detailed below and summarized in Table 2 and Fig. 1b.

**Table 2:** The recent clinical trials in ICI treatment for gastric cancer

Trial	Phase	Treatment target	Intervention	Control	Endpoint	Result
ATTRACTION-2	III	Progressive/ recurrent	Nivolumab monotherapy	Placebo	OS	Positive
ATTRACTION-4	III	Progressive/ recurrent (HER2^-^)	Nivolumab + chemo	Chemo	OS, PFS	Positive
CheckMate649	III	Progressive/ recurrent	Nivolumab + chemo	Chemo	OS, PFS (CPS > 5)	Positive
KEYNOTE-859	III	Progressive/ recurrent (HER2^-^)	Pembrolizumab + chemo	Chemo	OS	Positive
ATTRACTION-5	III	Adjuvant	Nivolumab + chemo	Placebo + chemo	RFS	Negative
KEYNOTE-585	III	Neo adjuvant/ adjuvant	Pembrolizumab + chemo/ Pembrolizumab	Placebo + chemo	OS, EFS, pCR rate	Negative
MATTERHORN	III	Neo adjuvant/ adjuvant	Durvalumab + chemo/ Durvalumab	Placebo + chemo	EFS	In progress
KEYNOTE-811	III	Progressive/ recurrent (HER2^+^)	Pembrolizumab + Trastuzumab + chemo	Placebo + Trastuzumab + chemo	OS, PFS	Positive
LEAP-015	I/II	Progressive/ recurrent (HER2^-^)	Lenvatinib + Pembrolizumab + chemo	–	Tolerability	In progress

Note: OS, Overall Survival; CPS, Combined Positive Score; PFS, Progression Free Survival; RFS, Recurrence-Free Survival; EFS, Event-Free Survival; pCR, pathological Complete Response.

In the treatment of gastric cancer, the effectiveness of ICI and ICI + chemotherapy for unresectable advanced recurrent cancer has been proven in multiple trials. On the other hand, negative results have been reported for the introduction of ICI perioperatively for resectable gastric cancer. As a new development, the effectiveness of ICI as neoadjuvant chemotherapy is being further explored, and the therapeutic effect of the combination therapy of molecular targeted drugs and ICI for HER2-positive gastric cancer is attracting attention.

ATTRACTION-2 study is an international phase III study that evaluated the efficacy and safety of in patients with advanced or metastatic gastric cancer. This study was a multicenter randomized study comparing nivolumab with placebo in patients who were resistant to chemotherapy or who progressed after chemotherapy. The results showed that the nivolumab group significantly prolonged OS compared with the placebo group (median 5.26 months in the nivolumab group vs. 4.14 months in the placebo group, HR = 0.63, 95% CI: 0.51–0.78, *p* < 0.0001). 12-month overall survival rates were 26.2% with nivolumab and 10.9% with placebo. Grade 3 or 4 TRAEs occurred in 10% of the nivolumab group and 4% of the placebo group. Deaths due to TRAEs occurred in 2% of the nivolumab group and 1% of the placebo group. In addition, the main side effects were fatigue, skin rash, and loss of appetite, and although immune-related side effects were sometimes observed, they were safely manageable [41]. The results of the ATRACTION-2 study led to the introduction of nivolumab monotherapy as a back-line treatment for gastric cancer.

ATTRACTION-4 study is a phase III trial evaluating whether the combination of nivolumab and chemotherapy (TS-1 + oxaliplatin or capecitabine + oxaliplatin) is more effective than conventional chemotherapy alone. The trial was conducted in Asia for patients with Human Epidermal Growth Factor Receptor 2 (HER2)-negative advanced or metastatic gastric cancer. The primary endpoints were OS and PFS in the entire patient population. As a final result, the combination therapy group significantly extended PFS compared to the chemotherapy alone group. However, no statistically significant difference in OS was observed between the two groups (*p* = 0.26). The median PFS was 10.45 months in the nivolumab plus chemotherapy group and 8.34 months in the placebo plus chemotherapy group (HR = 0.68, 98.51% CI: 0.51–0.90, *p* = 0.0007), and the median OS was 17.45 months in the nivolumab plus chemotherapy group and 17.15 months in the placebo plus chemotherapy group (HR = 0.90, 95% CI: 0.75–1.08, *p* = 0.26). The most common grade 3–4 TRAEs were neutropenia (20% in the nivolumab plus chemotherapy group vs. 16% in the placebo plus chemotherapy group) and platelet count decreased (9% vs. 9%). 6 treatment-related deaths occurred: 3 in the nivolumab plus chemotherapy group (one each due to febrile neutropenia, hepatic failure, and sudden death) and 3 in the placebo plus chemotherapy group (one each due to sepsis, hemolytic anemia, and interstitial lung disease). Regarding safety, immune-related side effects (e.g., pneumonia and hepatitis) were increased in the combination therapy group compared with the chemotherapy alone group, but were manageable with preventive measures [42].

CheckMate649 is an international phase III multicenter randomized clinical trial evaluating the efficacy of nivolumab plus chemotherapy in advanced gastric or gastroesophageal junction cancer. The chemotherapy regimen to be combined with nivolumab was selected to be capecitabine + oxaliplatin or FOLFOX (Fluorouracil + Levofolinate + Oxaliplatin). The primary endpoint of this study was OS and PFS in patients with high PD-L1 expression (CPS ≥ 5). The results showed that the combination of nivolumab and chemotherapy significantly extended OS compared with chemotherapy alone. The median OS in the nivolumab + chemotherapy group was 13.1 months, while the median OS in the chemotherapy alone group was 11.1 months, which was significantly extended (HR = 0.71, 98.4% CI: 0.59–0.86, *p* < 0.0001). PFS was also significantly improved (HR = 0.68, 98% CI: 0.56–0.81, *p* < 0.0001). Grade 3–4 TRAEs were observed in 59% of patients receiving nivolumab and chemotherapy and 44% of patients receiving chemotherapy alone. Deaths were considered treatment-related in 2% of patients receiving nivolumab and chemotherapy and 1% of patients receiving chemotherapy alone. Immune-related side effects such as pneumonia and hepatitis were observed, but few were fatal and could be safely managed [19]. Based on the results of the ATRACTION-4 and CheckMate649 studies, the combination of nivolumab and chemotherapy is indicated as first-line chemotherapy for HER2-negative gastric cancer.

KEYNOTE-859 study is a multicenter randomized phase III study evaluating the efficacy and safety of the combination of pembrolizumab and chemotherapy for HER2-negative advanced gastric or gastroesophageal junction cancer. This study compared the efficacy of pembrolizumab in combination with chemotherapy with chemotherapy plus placebo, and chemotherapy regimens could be selected from FP and capecitabine + oxaliplatin. The primary endpoint was OS. The results showed that the median OS in the pembrolizumab + chemotherapy combination group was 12.9 months compared to 11.5 months in the chemotherapy + placebo group, with the pembrolizumab combination group showing a significant improvement in OS (HR = 0.78, 95% CI: 0.70–0.87, *p* < 0.0001). In particular, in patients with high PD-L1 expression (CPS ≧ 10), the median OS in the pembrolizumab combination group was 15.7 months, while the median OS in the chemotherapy + placebo group was 11.8 months, showing greater therapeutic effects in this subgroup (HR = 0.65, 95% CI: 0.65–0.84, *p* < 0.0001), though this was a secondary analysis and should be interpreted accordingly. Serious TRAEs occurred in 23% of patients in the pembrolizumab group and 19% in the placebo group, and treatment-related deaths occurred in 1% of patients in the pembrolizumab group and 2% of patients in the placebo group. Regarding side effects, common immune-related side effects (skin rash, gastrointestinal disorders) were observed, but most were mild to moderate and could be safely managed [43].

Based on the results of the KEYNOTE-859 trial, the combination of pembrolizumab and chemotherapy is recommended as first-line chemotherapy for HER2-negative gastric cancer. However, there is no specific consensus on whether nivolumab or pembrolizumab should be used as a PD-1 antibody in first-line chemotherapy for HER2-negative gastric adenocarcinoma. In addition, there is no consensus on the chemotherapy regimen to be used in combination with PD-1 antibodies among studies, and there is no consensus. Furthermore, zolbetuximab has recently been shown to be effective against HER2-negative, Claudin (CLDN)-positive gastric cancer [44,45], and further discussion is needed regarding treatment strategies for HER2-negative, CLDN-positive gastric cancer with low PD-L1 expression.

On the other hand, the ATTRACTION-5 study was planned as a randomized phase III trial to explore the efficacy of nivolumab as adjuvant chemotherapy for gastric or gastroesophageal junction cancer. This study, planned in Asia, enrolled patients with pStage 3 gastrectomy and randomly assigned them to either the adjuvant chemotherapy + nivolumab group or the adjuvant chemotherapy + placebo group. The chemotherapy regimen was selected from TS-1 monotherapy or capecitabine + oxaliplatin, and the primary endpoint was RFS. The results showed that the 3-year RFS rate in the nivolumab + chemotherapy group was 64.8%, while the 3-year RFS rate in the placebo + chemotherapy group was 65.3% (HR = 0.90, 95% CI: 0.69–1.18, *p* = 0.4363), and the primary efficacy endpoint was not met [46]. The results of the ATTRACTION-5 trial did not demonstrate the superiority of nivolumab plus chemotherapy as postoperative adjuvant chemotherapy for gastric cancer and esophagogastric junction cancer, and ICI has not been introduced in postoperative adjuvant chemotherapy for gastric adenocarcinoma.

In addition, the KEYNOTE-585 study was conducted to investigate the additional effect of adding pembrolizumab therapy before and after surgery for gastric cancer or esophagogastric junction adenocarcinoma. Patients were randomly assigned to either a group that added pembrolizumab to neoadjuvant therapy or a group that added a placebo, and after radical resection, they received postoperative adjuvant therapy with the same regimen as before surgery. After four courses of postoperative adjuvant therapy, chemotherapy was removed and pembrolizumab alone or placebo was administered. The primary endpoints were pCR rate, EFS, and OS. The pCR rate was 12.9% in the pembrolizumab group compared to 2.0% in the placebo group, indicating that pembrolizumab was superior (*p* < 0.00001). EFS was also longer in the pembrolizumab group than in the placebo group, but did not meet the threshold for statistical significance (44.4 months vs. 25.3 months, HR = 0.81, 95% CI: 0.67–0.99, *p* = 0.0198). Moreover, OS was 60.7 months in the pembrolizumab group and 58.0 months in the placebo group, with no significant difference (HR = 0.90, 95% CI: 0.73–1.12, *p* = 0.174), and none of the primary endpoints were formally met apart from the pCR rate [47]. The results of the KEYNOTE-585 study concluded that the added effect of preoperative and postoperative pembrolizumab therapy on the prognosis of gastric cancer was negative.

The results of the ATTRACTION-5 and KEINOTE585 studies concluded that the significance of ICI in perioperative therapy for gastric adenocarcinoma was negative, and ICI has not been introduced into perioperative treatment for gastric adenocarcinoma.

### 3.2 Future Perspectives of ICI for Gastric Cancer

The future prospects for cancer immunotherapy using ICI in the field of gastric cancer can be divided into the introduction of ICI in perioperative chemotherapy and the development of ICI combination therapy aimed at increasing the therapeutic effect for unresectable advanced cancer. As mentioned above, there have been negative clinical trial results for the introduction of ICI in perioperative therapy, but clinical trials are underway to seek further possibilities. Representative clinical trial attempts are outlined below.

The MATTERHORN study is a randomized, double-blind, placebo-controlled phase III study to explore the therapeutic effects of preoperative FLOT plus the novel anti-PD-L1 antibody durvalumab in resectable gastric cancer and esophagogastric junction cancer. Patients are randomly assigned to either the FLOT + durvalumab group or the FLOT + placebo group before surgery, and in the durvalumab group, postoperative adjuvant chemotherapy with durvalumab alone is also added [48]. The primary endpoint is EFS. An interim report was presented at the conference showing that the pCR rate in the FLOT + durvalumab group was better than that in the FLOT + placebo group, but the primary endpoint and OS have yet to be reported. The results of the KEYNOTE-585 study also showed that the ICI combination group showed a good pCR rate, but no improvement in OS was observed, so the results of the MATTERHORN study also require careful interpretation.

As part of the development of ICI combination therapy aimed at increasing the therapeutic effect of unresectable advanced gastric cancer, the KEYNOTE-811 study was conducted to evaluate the effect of adding pembrolizumab to trastuzumab + chemotherapy, which is the gold standard for HER2-positive gastric cancer. Patients with HER2-positive gastric adenocarcinoma and esophagogastric junction adenocarcinoma were randomly assigned to either a group in which pembrolizumab was added to trastuzumab + chemotherapy or a placebo group. The combined chemotherapy regimen could be selected from FP or capecitabine + oxaliplatin, and the primary endpoints were PFS and OS. Although a significant improvement in PFS with the addition of pembrolizumab was reported, a significant improvement in OS was not easily achieved. It was finally reported that a significant improvement in OS was achieved in the final analysis [49]. In addition, differences in therapeutic effect depending on the degree of PD-L1 expression have also been reported, and it is suggested that, in the future, confirmation of PD-L1 status may become important in HER2-positive gastric cancer in light of these results.

The LEAP-015 trial is currently underway, which adds the tyrosine kinase inhibitor lenvatinib to pembrolizumab + chemotherapy as an attempt to develop a treatment for HER2-negative gastric cancer. FP and FOLFOX can be selected as chemotherapy regimens. It has been reported that safety has been confirmed in Part 1. Cases are continuing to accumulate in Part 2, which evaluates efficacy with OS and RFS as the primary endpoints [50].

The relationship between tumor-infiltrating lymphocytes (TILs) and cancer immunotherapy has also attracted attention in the field of gastric cancer, and various types of TILs that make up the tumor environment have been reported. Tumor-infiltrating monocytic myeloid-derived suppressor cells (TI-M-MDSCs) play a predominant role in the development of the immunosuppressive and ICI-resistant GC tumor immune microenvironment (TIME) [51]. TILs have also attracted attention in cancers other than gastric cancer, and it has been reported that the constituent cells of various TILs are deeply related to cancers [52].

## 4 Colon Cancer

### 4.1 Established Clinical Treatment Based on ICI for Colon Cancer

It is currently recognized that the efficacy of cancer immunotherapy in colon cancer depends on mismatch repair status. Immunostaining to examine the expression of MLH1, PMS2, MSH2, and MSH6 is commonly used to evaluate microsatellite instability (MSI) status. PCR and next-generation sequencing (NGS) are effective for detecting MSI, and NGS in particular allows for highly accurate MSI evaluation using only tumor tissue. These methods play an important role in screening for Lynch syndrome and determining the suitability of immune checkpoint inhibitors [53,54]. Colon cancer with MSI-high or DNA Mismatch Repair deficiency (dMMR) accounts for approximately 15% of all colon cancers, but is relatively rare at approximately 5% of advanced or metastatic cases. MSI-high colon cancer is histologically characterized as poorly differentiated histological types such as mucus-producing adenocarcinoma and signet ring cell carcinoma, and has been reported to occur predominantly in the right colon, particularly from the cecum to the ascending colon [55]. However, this group of tumors is characterized by an increase in TILs and activation of the PD-1/PD-L1 pathway, making it a group in which ICIs are expected to be effective [56,57]. However, the efficacy of monotherapy with PD-1 or PD-L1 inhibitors for microsatellite stable (MSS) colon cancer has not been demonstrated in multiple clinical trials. The recent clinical trials in ICI treatment for colon cancer are detailed below and summarized in Table 3 and Fig. 1c.

**Table 3:** The recent clinical trials in ICI treatment for colorectal cancer

Trial	Phase	Treatment target	Intervention	Control	Endpoint	Result
KEYNOTE-177	III	Progressive/ recurrent (MSI-H)	Pembrolizumab monotherapy	Chemo	OS, PFS	Positive
CheckMate142	II	Progressive/ recurrent (MSI-H)	Nivo monotherapy/ Nivolumab + Ipilimumab	–	OS, PFS, ORR	Positive
CheckMate8HW	III	Progressive/ recurrent (MSI-H)	Nivo monotherapy/ Nivolumab + Ipilimumab	Chemo	PFS	In progress
CheckMate 9X8	III	Progressive/ recurrent (MSS)	Nivolumab + chemo	Chemo	PFS	Negative
RELATIVITY-123	III	Progressive/ recurrent (MSS)	Nivolumab + Relatlimab (late line)	Chemo	OS	Negative
KEYFORM-007	III	Progressive/ recurrent (MSS)	Pembrolizumab + Favezelimab (late line)	Chemo	OS	Negative
LEAP-07	III	Progressive/ recurrent (MSS)	Pembrolizumab + Lenvatinib (late line)	Chemo	OS	Negative
NICHE-2	II	Neo-adjuvant (MSI-H)	Nivolumab + Ipilimuma	–	Tolerability	Positive
NICHE-3	II	Neo-adjuvant (MSI-H)	Nivolumab + Relatlimab	–	Tolerability	Positive
AZUR-2	III	Neo-adjuvant (MSI-H)	Dostarlimab monotherapy	Chemo	OS	In progress

Note: MSI-H, microsatellite instability-high; MSS, Microsatellite Stable; ORR, Objective Response Rate; OS, Overall Survival; PFS, Progression Free Survival.

In the treatment of colorectal cancer, the effectiveness of ICI differs significantly depending on the status of microsatellite instability. In the MSI-high group, the high efficacy of ICI has been proven, whereas the efficacy of ICI is negative in the MSS group. As a new development, the efficacy of ICI as neoadjuvant chemotherapy in the MSI-high group is being explored.

KEYNOTE-177 is a randomized, open-label, phase III study comparing pembrolizumab monotherapy with standard chemotherapy (FOLFOX or FOLFIRI (Fluorouracil + Levofolinate + Irinotecan) ± bevacizumab or cetuximab) in patients with previously untreated advanced MSI-high/dMMR colon cancer. Patients receiving standard chemotherapy were allowed to crossover to pembrolizumab up to 35 treatment cycles after progression. The primary endpoints were PFS and OS. As a result, the median RFS in the pembrolizumab group was 16.5 months, while the median RFS in the standard chemotherapy group was 8.2 months, demonstrating a significant prolongation of RFS in the pembrolizumab combination group (HR = 0.60, 95% CI: 0.45–0.80, *p* = 0.0002) [58]. In the final analysis, median overall survival was not reached for pembrolizumab vs. 36.7 months for chemotherapy, but the pre-specified alpha of 0.025 required for statistical significance was not reached, so superiority of pembrolizumab over chemotherapy in OS was not statistically demonstrated, although a favorable trend was observed (HR = 0.74, 95% CI: 0.53–1.03, *p* = 0.036) [59]. Grade 3 or higher treatment-related adverse events occurred in 22% of patients in the pembrolizumab group vs. 66% in the chemotherapy group (including one patient who died), showing a low incidence in the pembrolizumab group. The results of the KEYNOTE-177 study established pembrolizumab as a first-line treatment for untreated MSI-high/dMMR colon cancer.

CheckMate142 study is a multicenter, non-randomized, single-arm, phase II study of nivolumab and nivolumab + ipilimumab in patients with MSI-high/dMMR, previously treated with chemotherapy, who have unresectable advanced colon cancer. The primary endpoint was ORR, with PFS, OS, and safety as secondary endpoints. The ORR for the nivolumab monotherapy group was 31%, while the nivolumab + ipilimumab combination group reported favorable results of 55%. The nivolumab + ipilimumab combination therapy had a very high 12-month PFS of 71% and 12-month OS of 85%, confirming long-term disease control [60]. Grade 3–4 TRAEs were observed in 32% of patients, and any grade TRAEs led to treatment discontinuation in 13% of patients. Following the results of the CheckMate142 study, nivolumab and nivolumab + ipilimumab combination therapy have been introduced for patients with MSI-high/dMMR, previously treated with chemotherapy, who have unresectable advanced colon cancer.

Furthermore, the CheckMate8HW study, a randomized phase III study, is currently underway in response to the results of the CheckMate142 study. This study design compares three arms of nivolumab + ipilimumab, nivolumab alone, and standard chemotherapy (FOLFOX or FOLFIRI + bevacizumab or cetuximab can be combined) as first-line chemotherapy for unresectable advanced colon cancer with MSI-high/dMMR. The primary endpoint is PFS. Interim analysis results show that the PFS of nivolumab + ipilimumab is far superior to that of the standard chemotherapy group. Although the final study results are awaited, it is expected that nivolumab + ipilimumab will be available as first-line chemotherapy for unresectable advanced colon cancer with MSI-high/dMMR in the future [61].

On the other hand, negative results have been reported regarding the introduction of ICI for MSS colon cancer. In the CheckMate 9X8 study, which explored the effect of adding nivolumab to chemotherapy as first-line chemotherapy for MSS colon cancer, the PFS, which was set as the primary endpoint, did not meet the statistical significance threshold (11.9 months vs. 11.9 months, HR = 0.81, 95% CI: 0.53–1.23, *p* = 0.30) [62]. The RELATIVITY-123 study, which explored the efficacy of combination therapy of nivolumab and the novel anti-Lymphocyte Activation Gene 3 (LAG-3) antibody relatlimab as late-line chemotherapy for MSS colon cancer, also announced that the trial would be discontinued due to the expectation that the primary endpoint of OS would not be achieved [63]. Similarly, the KEYFORM-007 study, which investigated the efficacy of pembrolizumab + anti-LAG-3 antibody favezelimab combination therapy as late-line chemotherapy for PD-L1-positive MSS colon cancer, did not show a significant improvement in OS, its primary endpoint [64]. The LEAP-017 study, which investigated the efficacy of pembrolizumab + lenvatinib as third-line treatment for MSS colon cancer, also showed a trend toward an improvement in OS, its primary endpoint, but no statistically significant difference was reported [65].

### 4.2 Future Perspectives of ICI for Colon Cancer

As mentioned above, the efficacy of ICI for MSS colon cancer is presumed to be quite limited, and the development of new treatments, including further combination therapy, seems to be losing momentum. For unresectable advanced colon cancer with MSI-high/dMMR, ICI monotherapy or ICI doublet therapy has achieved very good treatment outcomes, and although further combination therapy is desirable, the priority of treatment development may be declining.

The focus of ICI-based treatment development for colon cancer is on perioperative treatment for resectable colon cancer with MSI-high/dMMR. The NICHE-2 trial demonstrated the efficacy of Nivolumab + Ipilimumab therapy for resectable dMMR colon cancer [66], and the NICHE-3 trial reported the efficacy of Nivolumab + relatlimab, an anti-LAG-3 antibody, for resectable dMMR colon cancer [67]. A phase II trial using the novel anti-PD-1 antibody dostarlimab as a neoadjuvant treatment for dMMR colon cancer has shown positive results [68]. Based on these results, the AZUR-2 trial is currently underway as an international phase III trial comparing dostarlimab with chemotherapy as neoadjuvant treatment for dMMR colon cancer.

These results suggest that ICI-based neoadjuvant chemotherapy may be highly effective for dMMR colon cancer, and there are high expectations for future treatment development.

## 5 Conversion Surgery after ICI-Based Treatment

Although treatment is still in the development stage, it is quite possible that an era will come in which unresectable advanced gastrointestinal cancers can be more effectively controlled with ICI-based treatment. As systemic tumor control is achieved through ICI-based treatment, the importance of local control of primary and metastatic lesions is increasing. Treatments that combine ICI-based treatment with local resection by surgery, so-called “conversion surgery” or local control by radiation therapy, are already being incorporated into clinical practice.

As a result of the recent advances in first-line treatment, including ICI for locally advanced/metastatic initially unresectable gastrointestinal cancer, surgery aiming at cure after initial treatment, so-called “conversion surgery” has become more common in this field [69,70]. In recent years, Chiba University has also experienced several cases in which local control was achieved by conversion surgery in addition to tumor control by systemic chemotherapy, including ICI.

**Figure 2:**
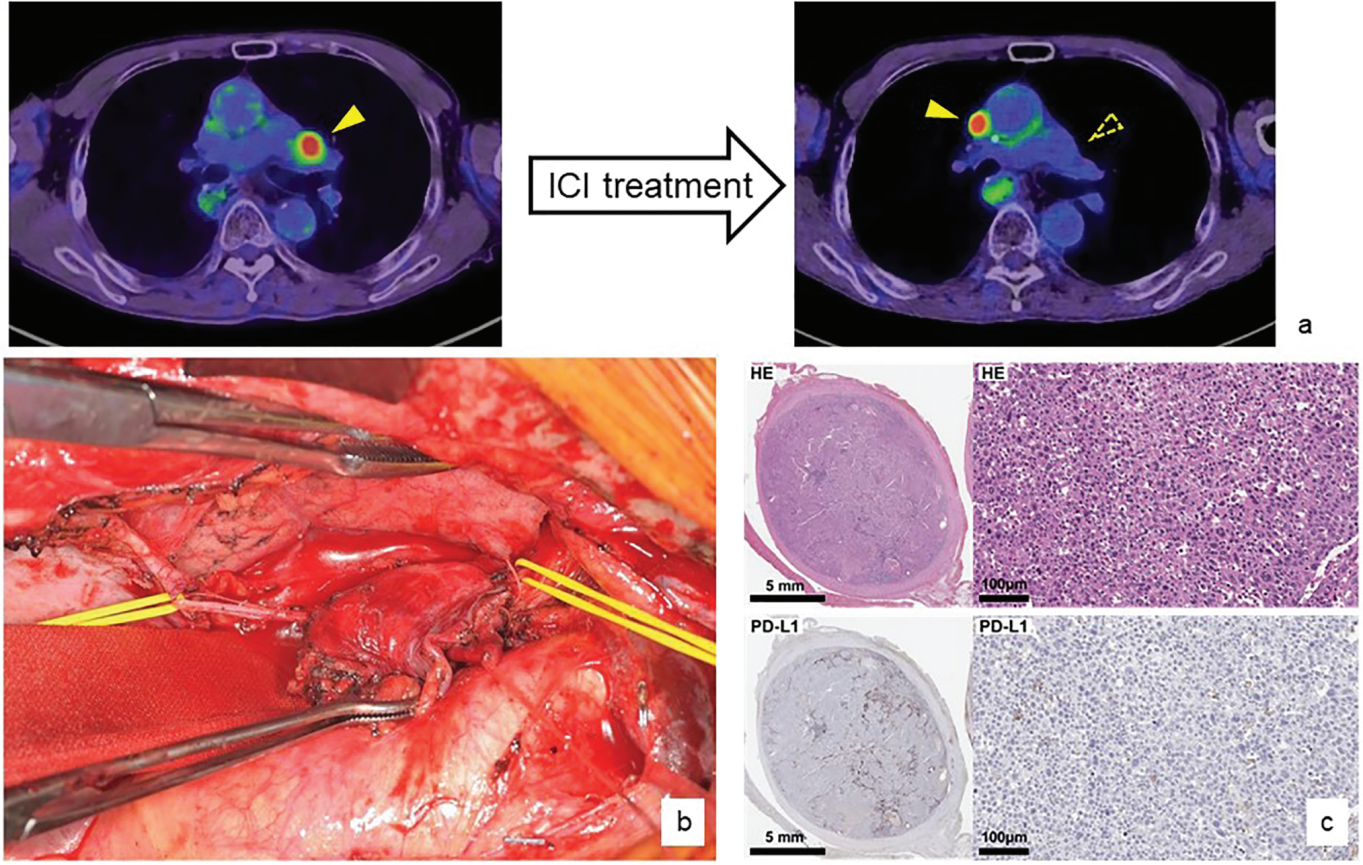
A specific example of conversion surgery after ICI treatment

A specific example of conversion surgery is the treatment of a 70-year-old male. He underwent radical resection following chemoradiotherapy for cStageIVA thoracic esophageal cancer, but anterior mediastinal lymph node recurrence occurred 9 months after surgery. cisplatin + 5-FU therapy followed by cancer immunotherapy using Nivolumab was successful, and the lymph node metastases disappeared, but another lymph node metastasis in contact with the ascending aorta remained. We determined that remission could be achieved by controlling these lymph node metastases, and therefore selected conversion surgery. The sternum was cut vertically to approach the local area, and the right phrenic nerve, which had been invaded by the tumor, was resected and the tumor was removed. After surgery, Nivolumab was continued as systemic chemotherapy, and although the patient died of other diseases due to rectal cancer, he achieved a disease-free survival for 2 years and 2 months from lymph node resection (Fig. 2).

A patient with anterior mediastinal lymph node recurrence from thoracic esophageal squamous cell carcinoma was treated with systemic chemotherapy, including nivolumab monotherapy. As a result, the lymph node metastasis disappeared and the patient was judged to have a complete response, but another anterior mediastinal lymph node metastasis appeared (a). A conversion lymphadenectomy was performed by splitting the sternum vertically. The lymph nodes had directly invaded the phrenic nerve (yellow taping), which required additional resection, but curative resection was possible (b). Pathological evaluation showed that the PD-L1 status of the resected metastatic lymph nodes was almost completely missing (c). Nivolumab monotherapy was continued after the conversion surgery and no recurrence or progression of esophageal squamous cell carcinoma was observed.

When conversion surgery is performed after ICI-based treatment and radical resection is clinically achieved, there is no consensus on whether additional treatment should be performed after surgery. An ICI-based treatment that was effective before surgery, or the administration of the ICI used at that time alone, may be a clinically effective option. On the other hand, follow-up without treatment is also an option, and in this case, there may be more options for re-administering ICI-based treatment at the time of recurrence. Further investigation is required to determine whether conversion surgery following ICI-based treatment is acceptable and whether additional treatment after surgery is recommended.

## 6 ICI-Based Treatment Strategy Based on Biomarkers

Treatment selection using biomarkers in ICI-based treatment for gastrointestinal cancers has not yet been established. In fact, biomarkers that accurately reflect the cancer immune status or the therapeutic effect of cancer immunotherapy have not yet been developed. This chapter describes the knowledge regarding treatment selection using biomarkers in gastrointestinal cancers to the extent that it is understood so far.

In the field of esophageal cancer, ICI-based treatment has not been selected based on biomarkers. Both the KEYNOTE-590 and CheckMate648 clinical trials have shown the effectiveness of ICI-based treatment regardless of the level of PD-L1 status, CPS and TPS. Therefore, ICI-based treatment is recommended for all cases regardless of the level of PD-L1 expression. There is no biomarker-based selection for which ICI-based treatment to select as the first line (FP + ICI or ICI doublet), or whether to use Nivolumab or Pembrolizumab.

**Figure 3:**
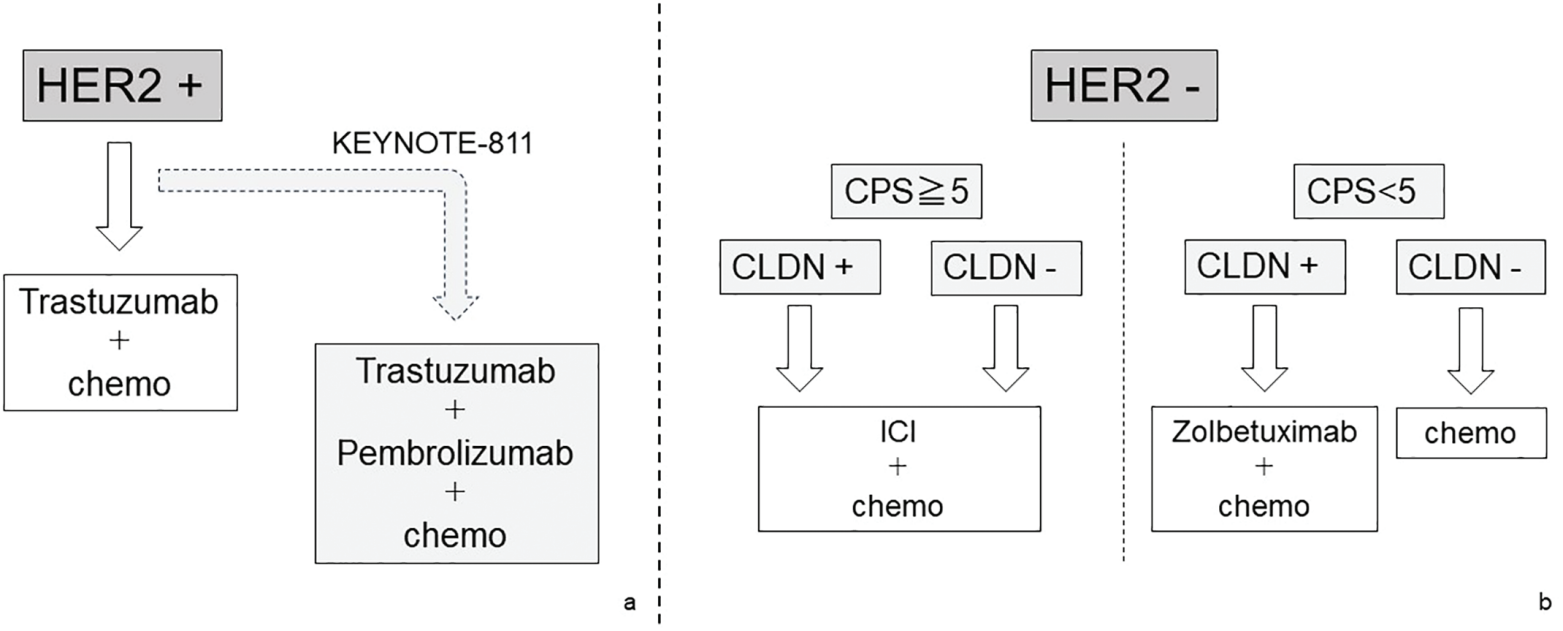
The schematic diagram of a treatment strategy for cStage IV gastric cancer

In the field of gastric cancer, the selection of a treatment strategy using biomarkers has become complicated. Prior to ICI-based treatment, molecular targeted therapy to HER2 using trastuzumab was introduced for HER2-positive gastric cancer. In recent years, zolbetuximab has been shown to be effective for Claudin-positive gastric cancer and has been introduced into clinical practice. There is no restriction on the indication for ICI-based treatment that requires confirmation of PD-L1 expression by CPS or TPS.　 However, for gastric cancer that is determined to be MSI-high, ICI-based treatment may be appropriate even if the cancer is found to be HER2 positive. From these complex situations, it is becoming essential to evaluate tumors using multiple biomarkers and select first-line chemotherapy in the field of gastric cancer. We summarize this complex situation in Fig. 3 and try to visualize it. Fig. 3 shows a schematic diagram of a treatment strategy for cStage IV gastric cancer inferred from current knowledge and future clinical trial results.

Treatment strategies for cStage IV and recurrent gastric cancer are divided by the HER2 expression status. In patients with positive HER2 expression, molecular targeted therapy for HER2 should be given top priority. Recently, the effectiveness of the combination therapy of HER2-targeted therapy and ICI has been explored (a). On the other hand, in patients with negative HER2 expression, CLDN expression and PD-1 expression should be searched for as additional biomarkers. In patients with high PD-1 expression, a treatment strategy including ICI is given priority, but for patients with negative PD-1 and positive CLDN, a regimen including Zolbetuximab should be selected. In patients with all negative HER2, PD-1, and CLDN, chemotherapy is one of the treatment options, but the effectiveness of ICI cannot be completely denied (b).

In the field of colon cancer, the effectiveness of ICI differs significantly depending on the status of microsatellite instability. In the MSI-high group, the high efficacy of ICI-based treatment has been proven, whereas the efficacy of ICI-based treatment is negative in the MSS group. In the treatment of colon cancer, the effectiveness of ICI-based treatment has not been demonstrated except in a small proportion of MSI-high patients, and conventional chemotherapy or molecular targeted therapy are chosen for the MSS group.

## 7 Conclusions

This article outlines the latest trends in ICI-based treatment in the field of gastrointestinal cancers. The expected therapeutic effects of ICI vary depending on the type of gastrointestinal cancer, and the direction of research and development is also completely different. In gastric cancers and colon cancers, ICI-based treatment is incorporated into the treatment plan selected using biomarkers, and it is expected that the treatment will become even more complicated in the future. At the same time, the development of more effective and sensitive biomarkers is also required. In both cancer fields, attempts to incorporate ICI into perioperative treatment and the development of further multidisciplinary treatments incorporating ICI are becoming a trend, and active treatment development is expected to continue in the future. Furthermore, in the near future, a treatment strategy that combines strict systemic control through ICI-based treatment with reliable local control through conversion surgery will become a powerful choice for gastrointestinal cancers. We should make optimal use of ICI-based treatment strategies and strive to further improve the prognosis of gastrointestinal cancers.

## Data Availability

Not applicable.
